# Highly Conductive Ionohydrogels for Humidity Sensing

**DOI:** 10.3390/polym17030327

**Published:** 2025-01-25

**Authors:** Min-Na Sun, Wen-Yu Chen, Li Wang, Zhi-Gang Wang, Lei Qin, Xu-Ming Xie

**Affiliations:** 1 Beijing Key Laboratory for Sensors, Beijing Information Science and Technology University, Beijing 100192, China; sunminna331@163.com (M.-N.S.); chenwenyu@bistu.edu.cn (W.-Y.C.); 2 Key Laboratory of Modern Measurement & Control Technology, Ministry of Education, Beijing Information Science and Technology University, Beijing 100192, China; 3 Beijing Key Laboratory for Optoelectronic Measurement Technology, Beijing Information Science and Technology University, Beijing 100192, China; 4 China Mobile Park Construction and Development Co. Ltd., Beijing 102200, China; 5 Key Laboratory of Advanced Materials of Ministry of Education (MOE), Department of Chemical Engineering, Tsinghua University, Beijing 100084, China

**Keywords:** ionohydrogel, humidity sensing, retention, highly conductive

## Abstract

Polymeric hydrogel materials have excellent electrical conductivity and mechanical properties and will be potentially used in wearable electronic devices, soft robotics, and medical treatment. In this paper, a PAA-Fe^3+^-IL ionohydrogel (poly(acrylic acid)-Fe^3+^-ionic liquid ionohydrogel) with excellent mechanical and conductive properties is prepared by simple free radical polymerization. The presence of metal-ligand crosslinking within the ionohydrogel improves the mechanical properties of the hydrogel. When the IL content is 10 wt%, it has the maximum tensile strength and strain. When the ferric ion concentration is 0.3 mol%, the maximum tensile strength is 495.09 kPa. When the ferric ion concentration is 0.1 mol%, the maximum strain is 1151.35%. The tensile behavior of the ionohydrogels is quantitatively analyzed by the viscoelastic model. In addition, free metal ions and anions and cations in IL endowed the hydrogel with a conductivity of 1.48 S/m and a strain sensitivity of 8.04. Thus, the PAA-Fe^3+^-IL ionohydrogel can be successfully used as a humidity sensor due to the hydrophilic ionic liquid, which can increase the conductivity of the hydrogel by absorbing water. The physical crosslinking density inside the hydrogel is much higher than the chemical crosslinking density, which causes hydrogel dissolution in deionized water by swelling and is conducive to the recycling of the hydrogel. This is a promising material for use in intelligent wearable electronics and as a humidity sensor.

## 1. Introduction

Hydrogels are well-known physically or chemically crosslinked hydrophilic polymers containing large amounts of water or biological fluids [1,2,3,4,5,6]. Due to these characteristics, in recent years, they have attracted the attention of many researchers in many fields, such as drug delivery systems, wastewater treatment, and agriculture [7,8,9,10,11]. It is of great significance to develop polymer hydrogels with excellent mechanical properties and conductivity [12,13,14,15,16,17].

Acrylic acid (AA) monomers are often used in the industrial production of hydrogels because of their low toxicity, good water absorption ability, and excellent biocompatibility and biodegradability. Warunee Tanan et al. [18,19,20,21,22,23] prepared a semi-interpenetrating polymer network hydrogel which was made by a kind of cassava starch-g-polyacrylic acid/natural rubber/polyvinyl alcohol blend, it showing good biodegradability [24,25,26,27,28]. While the amount of physical crosslinking is much greater than that of chemical crosslinking in the synthesized hydrogel, it has excellent degradable properties. The coordination between Fe^3+^ and the acrylic group shows that the obtained hydrogel has ultra-high mechanical properties and good self-recovery performance [29,30,31,32,33]. Generally, carboxyl-Fe^3+^ coordination bonds can be formed in situ free radical polymerization of acrylic acid (AA) and Fe^3+^ solution directly. However, excess amounts of Fe^3+^ (more than 1.0 mol%) will retard this free radical polymerization and reduce the molecular weight of polymer chains, which will decrease the mechanical properties of the hydrogel [34,35,36].

Currently, the multi-bond network (MBN) strategy has been shown to be a very effective method that can substantially improve the toughness of hydrogels [37,38,39,40]. A typical MBN gel has a single polymer network, which contains hierarchical physical or covalent crosslinks with different bonding energies. Upon the stretching, dynamic interactions, such as hydrogen bonding, ionic interactions, metal-ligand bonding, and hydrophobic conjugation, the bonds are fractured and the energy is dissipated gradually. The hydrogen bonds can recombine some new sites to form a more homogeneous network due to the reversible nature. For example, carboxyl-Fe^3+^ metal interactions with high bonding energies are widely used in hydrogel systems to improve their mechanical strength. The MBN strategy effectively improves the toughness of hydrogels, on the basis of which the improvement of electrical conductivity needs to be addressed.

Therefore, in order to obtain hydrogels with the intended tensile properties and conductive properties, in this work, ionohydrogels with a homogeneous network are prepared in ionic liquid, 1-ethyl-3-methylimidazole acetate, and ferric ions. The ferric ions formed metal-ligand crosslinking bonds with the carboxyl group of PAA chains, which can enhance the toughness of the ionohydrogels. A humidity sensing test was performed of the high-conductivity ionohydrogel to assess its performance as a humidity sensor.

## 2. Materials and Methods

### 2.1. Materials

Acrylic acid (AA), ionic liquid (IL) 1-ethyl-3-methylimidazole acetate, N, N′-methylene bisacrylamide (MBA), ammonium persulfate (APS), and ferric chloride hexahydrate were purchased from Macklin(Shanghai, China), and deionized water was used. AA needed to be distilled under reduced pressure to remove the barrier, and the rest of the sample was used directly without further purification.

### 2.2. Ionohydrogel Preparation

A PAA-Fe^3+^ MBN ionohydrogel was prepared by free radical polymerization. First of all, IL (0 wt%~15 wt%) and deionized water (55 wt%~70 wt%) were mixed. MBA, APS, and hexahydrated ferric chloride are solids, so these three substances needed to be added to deionized water first to form an aqueous solution. Then, AA (30 wt%) and a crosslinking agent were added to the mixture of IL and deionized water.

The mixed solution of MBA (0.0012 wt%), the initiator APS (0.012 wt%), and FeCl_3_-6H_2_O (0.5 mol%~1.5 mol%) was subjected to sonication to obtain a uniform mixed solution. Finally, bubbles were ultrasonically removed from the mixed solution. The prepolymer was put into a mold, it was sealed at 45 ℃ for 40 h. In this work, the ionohydrogel is referred to as PAA-Fe^3+^x-ILy, where x represents the concentration of Fe^3+^ and y indicates the content of IL in the system.

### 2.3. Performance Characterization

The morphology and microstructure of the sample were characterized by scanning electron microscopy (SEM).

Before the SEM test, it was necessary to remove the moisture, so the sample was frozen and freeze-dried. First, the ionohydrogel was placed in liquid nitrogen for freezing and brittle failure. The temperature of the liquid nitrogen at normal pressure was −196 ℃, which can easily freeze the water in the ionohydrogel, so the frozen ionohydrogel was then put into the freeze-dryer at a low temperature (−50 ℃) to freeze-dry in a vacuum (0.040 mbar). Finally, the cross-section of the obtained freeze-dried ionohydrogel was observed, and electron microscopy photos were obtained [38,41,42,43,44,45,46,47].

The mechanical performance test of the ionohydrogel was carried out using a Shimadzu AGS-X tensile testing machine (Shimadzu, Kyoto, Japan). The tensile test was performed using a cylindrical sample (4 mm in diameter), and the distance between the clamps of the sample was 15 mm. For the uniaxial tensile test, the tensile rate was 100 mm/min, and the stress-strain curve of the material was obtained (Fe^3+^ 1.0 mol%~2.0 mol%, IL 0 wt%~15 wt%). The tensile load, F, and displacement, x, were recorded [48,49,50,51,52,53].

The tensile stress was calculated as follows:

(1) σ=F/S

The tensile strain was calculated with the following formula:

(2) $$\varepsilon =x/l_{0}$$

The conductivity of the ionohydrogel was measured using an LCR digital bridge meter (UT622E, UNI-T, Changsha, China) to test the O-resistance value of the hydrogel sample (the length of the cylinder-shaped sample was 30 mm and the diameter was 3 mm; the length of the thin sample was 20 mm and the thickness was 1 mm), and this was calculated using Formula (3).

(3) K=L/SR

In this equation, L is the sample length, S is the cross-sectional area, and R is the resistance.

## 3. Results and Discussion

### 3.1. Structure and Characterization of PAA-Fe^3+^-IL Ionohydrogel

**Figure 1:**
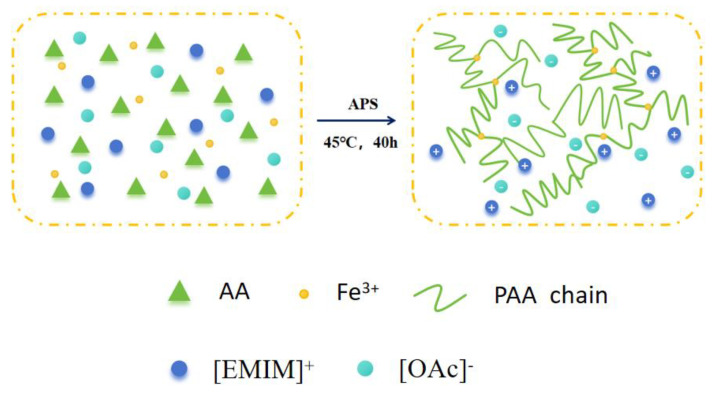
Illustration of the synthesis and network structure of the PAA-Fe^3+^-IL ionohydrogels.

PAA-Fe^3+^-IL ionohydrogels were prepared through a simple one-pot free radical polymerization process, in which the ionic liquid selected was 1-ethyl-3-methylimidazole acetate ([EMIM][OAc]) due to its hydrophilicity, strong hydrogen bonding, high electrical conductivity, and low freezing point. Ionohydrogels were prepared through the polymerization of monomers, using acrylic acid (AA) with N,N′-methylenebisacrylamide (MBA) and ammonium persulfate (APS) as a chemical crosslinking agent and thermal initiator, respectively. PAA-Fe^3+^-IL ionohydrogels were obtained due to the relatively good coordination ability of ferric ions. As shown in Figure 1, AA was linked to polyacrylic acid chains (PAA) under the action of the crosslinking agent and initiator, and a large number of metal-ligand crosslinks were directly formed in situ between Fe^3+^ and the carboxyl groups (-COO-) on the PAA chains.

**Figure 2:**
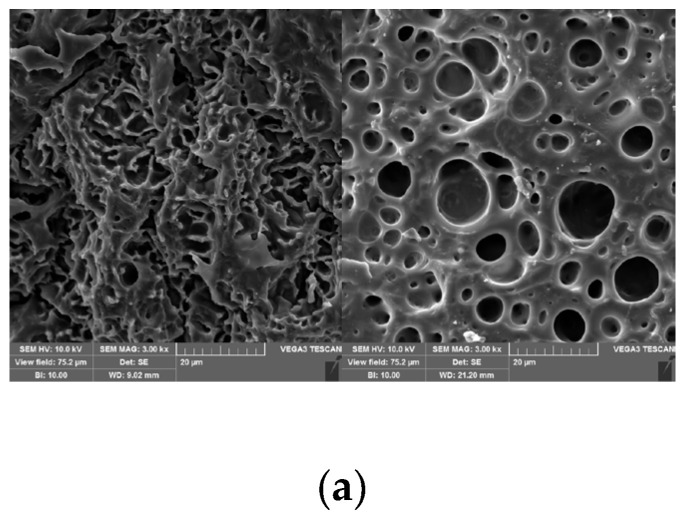

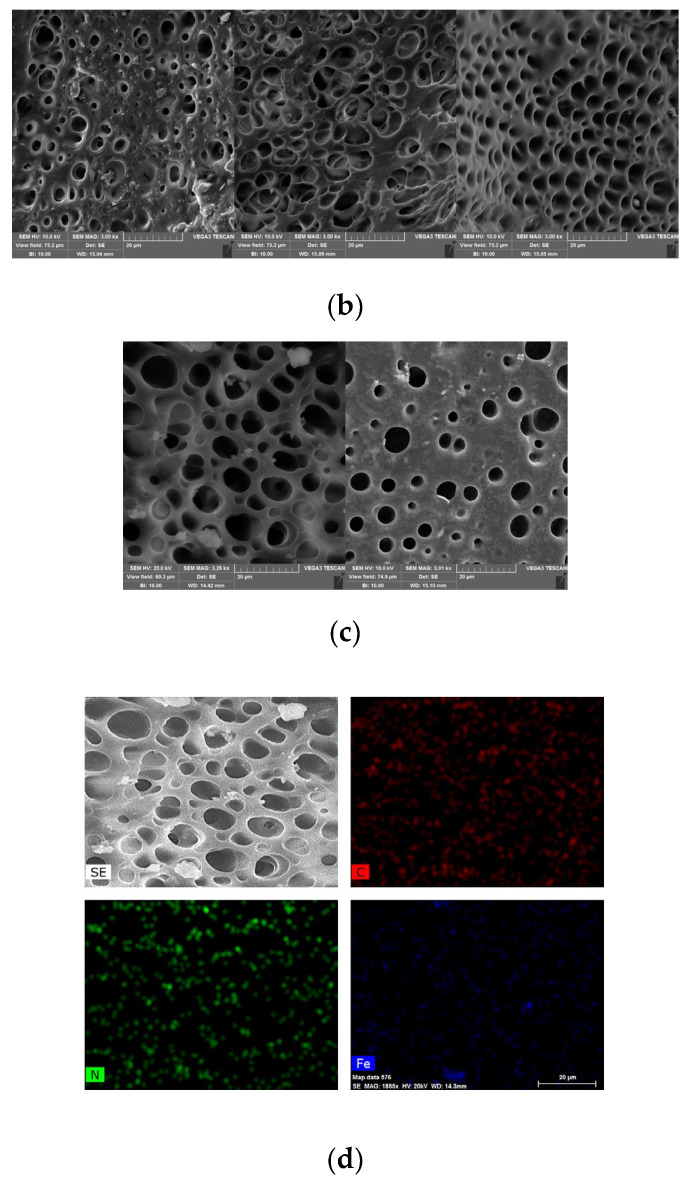
(**a**) SEM of ionohydrogels with 0.05 mol% and 0.1 mol% Fe^3+^ concentrations; (**b**) SEM of ionohydrogels with 0.3 mol% Fe^3+^ concentration; (**c**) SEM of ionohydrogels with 0.5 mol% Fe^3+^ concentration; (**d**) element mapping of elements in the dried PAA-Fe^3+^-IL ionohydrogel.

Figure 2a–d demonstrate the SEM micrographs and elemental maps of the lyophilized hydrogel. By observing Figure 2, it can be seen that the synthesized PAA-Fe^3+^-IL MBN ionohydrogel has a well-distributed network structure. The Fe^3+^ concentration and IL content (10 wt%) were observed at the same magnification (3kx). Electron microscopy images for different ferric ion concentrations and an IL content of 10 wt% can be observed. SEM photos of the ionohydrogels with 0.05 mol% (Figure 2a), 0.1 mol% (Figure 2a), 0.3 mol% (Figure 2b), and 0.5 mol% Fe^3+^ concentrations (Figure 2c) can be seen in Figure 2. It can be observed that the pore distribution in Figure 2c is the most even. This phenomenon indicated that the network crosslinks are evenly distributed. This phenomenon clearly shows that the ionohydrogel structure has an even pore-like structure, in which Fe and N are evenly distributed. In other words, the IL and ferric ions are evenly distributed in the PAA-Fe^3+^-IL ionohydrogel. This evenly crosslinked network will provide good conductivity.

Using EDX to observe the dispersion of the internal elements of the PAA-Fe^3+^-IL MBN ionohydrogel, the distribution of C, N, and Fe in the sample can be seen in Figure 2d. It is because that the crosslinking of the ferric ions and PAA formed the evenly network structures. Within this structure, more ferric ions are crosslinked with PAA as metal coordination bonds, and the ionic liquid is evenly distributed inside the ionohydrogel, which plays an important role in improving its electrical conductivity and mechanical properties.

### 3.2. Mechanical Properties of PAA-Fe^3+^-IL Ionohydrogel

#### 3.2.1. Experiment

**Figure 3:**
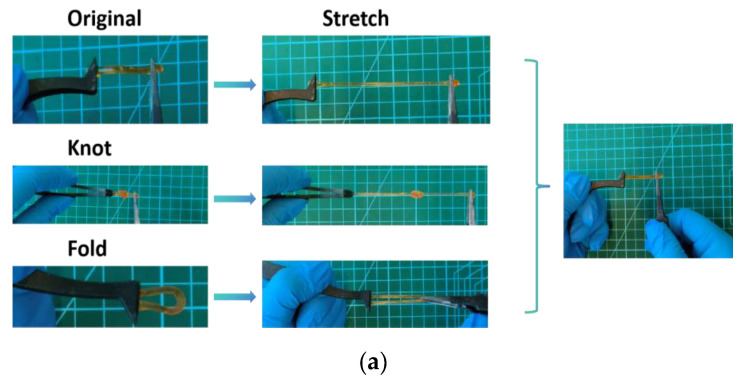

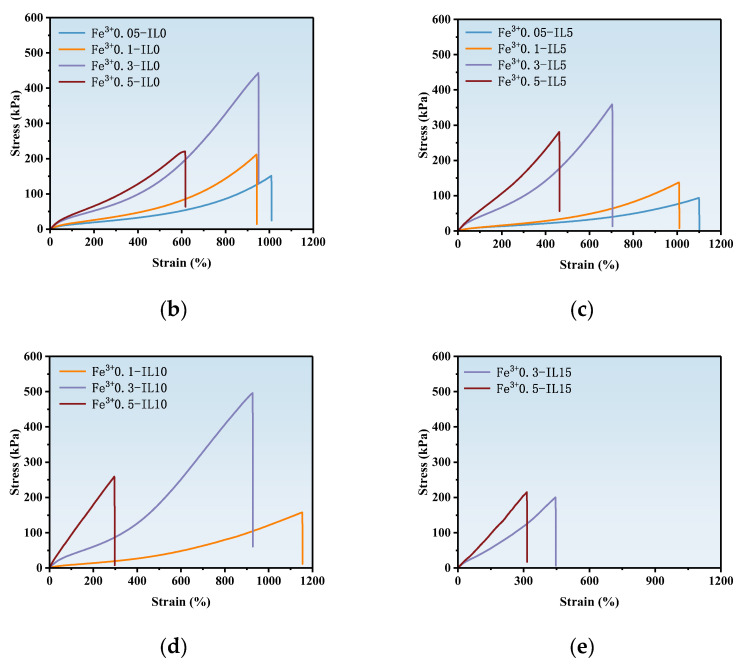
(**a**) PAA-Fe^3+^-IL MBN ionohydrogel stretching, knot stretching, and fold stretching status diagram. (**b**) Stress-strain curves for varying Fe^3+^ concentrations with a fixed IL content of 0. (**c**) Stress-strain curves for varying Fe^3+^ concentrations with a fixed IL content of 5 wt%. (**d**) Stress-strain curves for varying Fe^3+^ concentrations with a fixed IL content of 10 wt%. (**e**) Stress-strain curves for varying Fe^3+^ concentrations with a fixed IL content of 15 wt%. (The relative humidity and temperature for the experiment were 25 °C and 38 RH%).

The mechanical properties of the PAA-Fe^3+^-IL ionohydrogel was tested by stretching, knot stretching and fold stretching, as shown in Figure 3a. The initial state was restored after stretching. The experiment’s results indicate that it has excellent deformation ability. The mechanical properties of the PAA-Fe^3+^-IL MBN ionohydrogel were tested using a Shimadzu AGS-X elongation testing machine. The tensile test was carried out by the method of the Methods and Materials Section. Figure 3b,c shows the increase of the stress-strain curve with increasing the Fe^3+^ concentration (horizontal coordinates represent strain and vertical coordinates represent stress, respectively). The stress-strain of the Fe^3+^ concentrations of 0.05 mol% (light blue line), 0.1 mol% (orange line), 0.3 mol% (purple line), and 0.5 mol% (dark red line) were shown in Figure 3b,c. The PAA-Fe^3+^-IL MBN ionohydrogel shows excellent tensile performance. The tensile strength of the material was improved. A PAA-PAM-Fe^3+^ hydrogel with a double-crosslinked network was obtained in Yuntong Liu’s work, and a maximum tensile strength of 180 kPa [54]. Ionohydrogels have evenly distributed network structures, which give them excellent mechanical properties. When the IL content is 5 wt%, the tensile strength can reach up to 358 kPa at an Fe^3+^ concentration of 0.3 mol%. When IL is not added and the Fe^3+^ concentration is increased to 0.3 mol%, the tensile strength can reach up to 442.7 kPa.

When the content of IL is 10 wt%, at low Fe^3+^ concentrations, the ionohydrogel becomes brittle and cannot be stretched. When the concentration of Fe^3+^ is 0.1 mol%, it has a maximum strain of 1151.35%. When the Fe^3+^ concentration increases to 0.3 mol% and the IL content is 10 wt%, the tensile strength reaches the maximum of 495.09 kPa, as shown in Figure 3d. This is because that the appropriate IL content can promote the crosslinking of metal ions, which improve the mechanical properties of ionohydrogel. Whereas, the excessive rigid interlinking can reduce the flexibility of ionohydrogels.

When the IL content increases to 15 wt% and the concentration of Fe^3+^ is less than 0.1 mol/L, the mechanical properties are too weak to strain. The stress-strain curve is shown in Figure 3e (the purple line represents that the Fe^3+^ concentration is 0.3 mol%, and the dark red line represents that the Fe^3+^ concentration is 0.5 mol%). When the concentration of Fe^3+^ is increased, the tensile strength and fracture elongation will become weak. The maximum tensile strength is only 214.82 kPa, and the highest fracture elongation is only 445.79%.

After comparisons of the mechanical properties of PAA-Fe^3+^-IL MBN ionohydrogels with different Fe^3+^ concentrations and IL contents, the MBN ionohydrogel with an Fe^3+^ concentration of 0.3 mol% and an IL content of 10 wt% was selected. Its tensile strength was 495.09 kPa, and its elongation rate was 924.68%. Compared with other types of ionohydrogels, this fracture elongation is slightly reduced, but it can still meet the requirements of mechanical properties in practical applications. The mechanical properties of this ionohydrogel meet the requirements for use in electronic skin and provide ideas for the development of electronic skin.

The addition of IL and free ions to the system will increase the electrical conductivity of the ionohydrogel. The formation of strong hydrogen bonds between IL and water molecules prevented the evaporation and freezing of water molecules in the ionohydrogel. Therefore, the ionohydrogel has excellent tensile ability and conductivity with water retention and anti-freezing ability.

#### 3.2.2. Stress-Strain Curve Simulation Model

To evaluate the interaction of physical and chemical crosslinking inside the ionohydrogel and to explore the role of different bonds, such as hydrogen bonds and ionic bonds, in responding to changes in stress and strain, we introduce a constitutive model. Therefore, the dynamic molecular structure of these ionohydrogels is quantitatively elucidated for their stretching behavior.

A prediction of the uniaxial stress-strain curve can be extracted from the constitutive model, and it comes out as the sum of contributions to the stress from the UCM element and the Gent element, which can be written as follows:

(4) $$\sigma_{N}(\lambda )=\sigma_{N,v}(\lambda )+\sigma_{N,e}(\lambda )$$

where

(5) $$\sigma_{N,v}\left(\lambda \right)=\{\frac{2G_{v}D_{e}}{1-2D_{e}}\left[1-exp\left(-\frac{\left(1-2D_{e}\right)}{D_{e}}\left(\lambda -1\right)\right)\right]+\frac{G_{v}D_{e}}{1+D_{e}}\left[1-exp\left(-\frac{\left(1+D_{e}\right)}{D_{e}}\left(\lambda -1\right)\right)\right]\}\lambda^{-1}$$

and

(6) $$\sigma_{N,e}(\lambda )=[\frac{G_{e}}{1-\frac{\lambda^{2}+2/\lambda -3}{J_{m}}}(\lambda^{2}-1/\lambda )]\lambda^{-1}$$

where G_v_ is the initial shear modulus of the viscoelastic part, D_e_ (t_rel_) is the Deborah number (the product of the relaxation time of the viscous component and the strain rate), G_e_ is the small-strain shear modulus of the elastic part, J_m_ (mm) is the maximum allowable value of the first strain invariant, and λ is the extension ratio [55,56,57].

This model is a parallel combination of the upper convection Maxwell (UCM) model for describing viscoelastic flow and the Gent strain-hardening model of entropic elasticity for the finite elongation of polymer chains. The model captures the physical characteristics of the viscoelastic phase from the dynamic weak ionic bonding and strong bonding in the elastic phase through permanent crosslinking [58,59]. In other words, G_v_ reflects physical crosslinking, G_e_ and J_m_ reflect chemical crosslinking, and λ reflects the magnitude of the strain.

**Figure 4:**
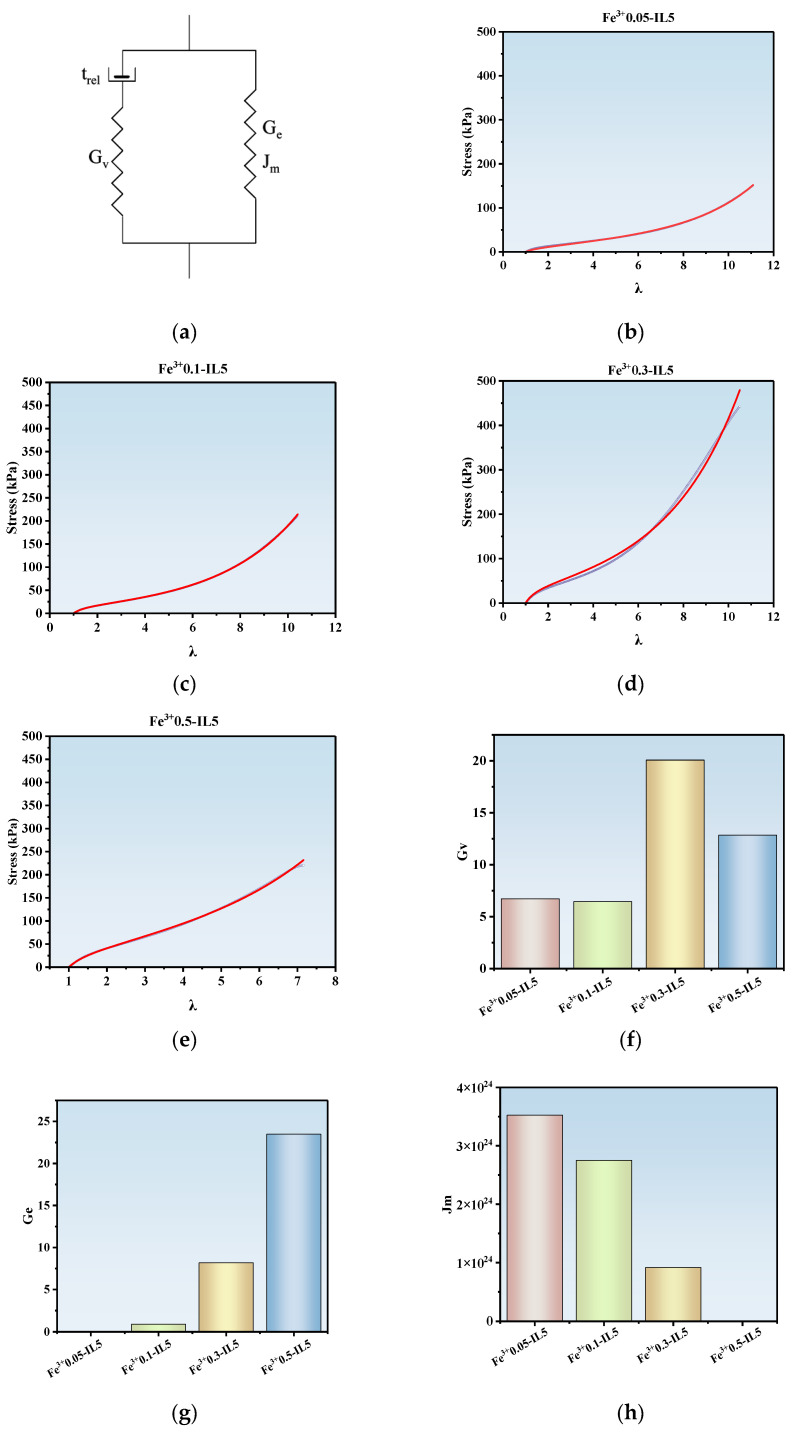
(**a**) Schematic of the non-linear viscoelastic model used. (**b**–**e**) Typical tensile behavior (experimental data) and sum of simulated contributions (theoretical fitting curves) of PAA-Fe^3+^-IL ionohydrogels at an IL content of 5 wt% and Fe^3+^ concentrations of 0.05 mol%~0.5 mol%, respectively. (**f**) The fitting data for the shear modulus of the viscoelastic phase, G_v_. (**g**) The fitting data for the shear modulus of the elastic phase, G_e_. (**h**) The fitting data for the theoretical finite extensibility, J_m_.

Figure 4a shows a schematic of the non-linear viscoelastic model used. The best fits of this model with the tensile curves shown in Figure 3 are presented in Figure 4b–e. Red lines are the fitted curves. Blue lines are the stretch test curves. It is obvious that a very good fit can be obtained to the data. The model fitting of the tensile behavior leads to the extraction of quantitative structural parameters, including the initial shear modulus, G_v_ (kPa), of the viscoelastic phase, the shear modulus, G_e_ (kPa), of the elastic phase, and the theoretical finite elongation of the network chains, J_m_. The given initial strain rate is 100 mm/min. The fitted data of G_v_, G_e_ and J_m_ (mm) are plotted in Figure 4f–h. When the IL content was fixed at 5 wt% and the Fe^3+^ concentration was increased to 0.3 mol%, physical crosslinking increased to the maximum. Meanwhile, the physical crosslinking disappeared when the Fe^3+^ concentration continued to increase. The chemical crosslinking decreased with increasing the concentration of ferric ions. From Figure 4h, it can be seen that J_m_ decreases by increasing the Fe^3+^ concentration monotonically. This is consistent with the stretching trend observed in the experiment.

#### 3.2.3. Sensitivity and Cyclic Stretching

The strain sensitivity of the ionohydrogel samples was quantitatively assessed by the gauge factor (GF) using the following equation:

(7) $$GF=(R-R_{0})/R_{0}\varepsilon$$

where R_0_ is the initial resistance of the ionohydrogel samples, R is the resistance at a certain strain, and ε refers to this strain.

**Figure 5:**
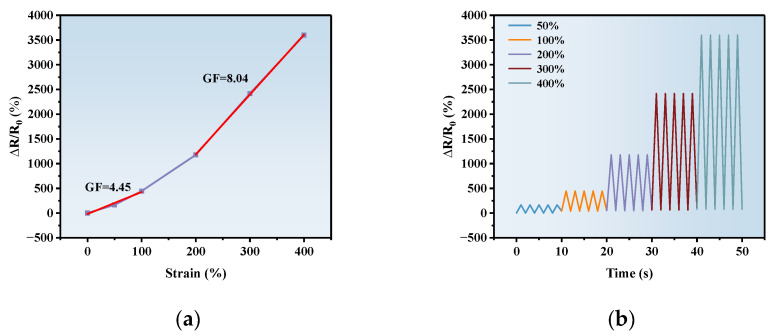
(**a**) Relative change in the resistance of the PAA-Fe^3+^-IL ionohydrogels relative to the tensile strain; (**b**) resistance variability of PAA-Fe^3+^-IL ionohydrogels at 50%, 100%, 200%, 300%, and 400% strain.

Electrical sensitivity is vital for the application of ionohydrogels in intelligent wearable electronics, and the gauge factor (GF) is often used to quantitatively assess the strain sensitivity of materials. Figure 5a shows the relative change in the resistance of the PAA-Fe^3+^-IL ionohydrogels relative to the tensile strain when the PAA content is 30 wt%, the IL content is 15 wt%, and the Fe^3+^ concentration is 0.5 mol%. Obviously, the sensitivity of the ionohydrogels relative to the tensile strain can be divided into two main regions. In the initial strain range of 0~100%, the GF of the ionohydrogels is estimated to be 4.45, while when the strain is more than 100%, the GF is increased to 8.04. With increasing tensile strain, the migration distance of the ions in the ionohydrogels becomes longer, which leads to the resistance of the ionohydrogels increasing. Hence, the value of the GF is enhanced. Moreover, highly conductive and sensitive PAA-Fe^3+^-IL ionohydrogels respond rapidly to different strains repeatedly, as shown in Figure 5b. These promising materials will be used in intelligent and wearable strain sensors.

### 3.3. Conductivity of PAA-Fe^3+^-IL Ionohydrogels

**Figure 6:**
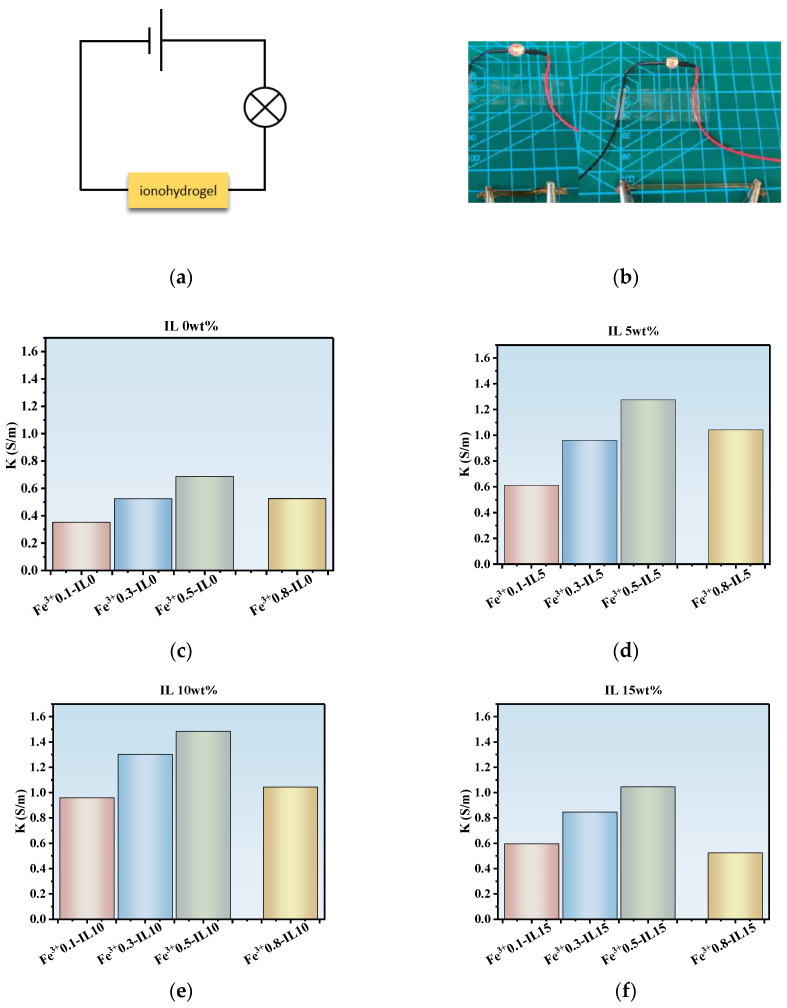
(**a**) Schematic diagram of ionohydrogel’s access to conductive pathway. (**b**) Brightness of ionohydrogel in original state and after stretching. (**c**) Variation in conductivity with Fe^3+^ concentration at a fixed IL content of 0 wt%. (**d**) Variation in conductivity with Fe^3+^ concentration at a fixed IL content of 5 wt%. (**e**) Variation in conductivity with Fe^3+^ concentration at a fixed IL content of 10 wt%. (**f**) Variation in conductivity with Fe^3+^ concentration at a fixed IL content of 15 wt%.

The PAA-Fe^3+^-IL ionohydrogel has excellent electrical conductivity due to the presence of a mount of the anions and cations in the system. Figure 6a,b shows the ionohydrogel connected to LEDs. The fact that these LEDs still glow proves the existence of electrical conductivity. When the channel connected to the ionohydrogel is stretched, the longer the stretching distance of the ionohydrogel, the darker the brightness of the bulb. When strain is applied to the ionohydrogel, it can be seen that the light becomes weaker. When the applied strain is removed, the light immediately becomes brighter. This is because the ionohydrogel was stretched, this lead to the free ions moving need longer distance. Therefore, the resistance of the ionohydrogel is increased.

The conductivity of the PAA-Fe^3+^-IL MBN ionohydrogel at different Fe^3+^ concentrations (0.1 mol%~0.8 mol%) is shown in Figure 6c–f. When the IL content is 10 wt% at room temperature, with increasing Fe^3+^ concentration, the conductivity of the ionohydrogel increases from 0.95 S/m to nearly 1.48 S/m, as shown in Figure 6e. When the IL content is 15 wt%, with increasing Fe^3+^ concentration to 0.5 mol%, the conductivity of the ionohydrogel increases from 0.59 S/m to 1.04 S/m, as shown in Figure 6f. When the Fe^3+^ concentration is 0.5 mol%, the conductivity is the highest. When the Fe^3+^ concentration continues to increase, the conductivity begins to decline. This is because the crosslinking density of ferric ions is increased with increasing IL content; meanwhile, the free-moving ions are constrained. When the IL concentration is higher, the speed of free-moving ions and the conductivity will decrease. An appropriate concentration of Fe^3+^ can improve the conductivity. This is because the ferric ions as the mobile ions will increase the conductivity. An excessive number of ferric ions will disrupt the network, resulting in a slight decrease in conductivity. This excellent conductivity provides the necessary conditions for humidity sensing, giving the material excellent humidity sensing capabilities.

### 3.4. Degradation of PAA-Fe^3+^-IL Ionohydrogels

The swelling performance and water absorption of the ionohydrogel were tested at room temperature. Firstly, the weighed ionohydrogel was soaked in a large amount of deionized water. Secondly, after the ionohydrogel was soaked in deionized water for 10 min, it was taken out, the water on the ionohydrogel’s surface was removed, and it was weighed.

Through the swelling test, the water absorption rate was calculated. The calculation formula is

(8) $$Q=(m-m_{0})/m_{0}\times 100\%$$

In this equation, m is the weight after water absorption and m_0_ is the initial weight.

**Figure 7:**
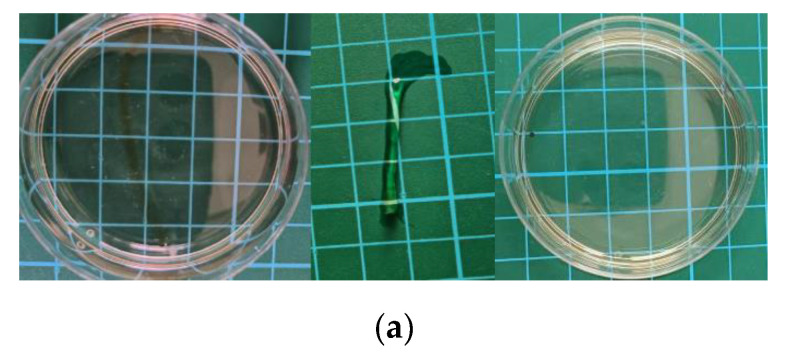

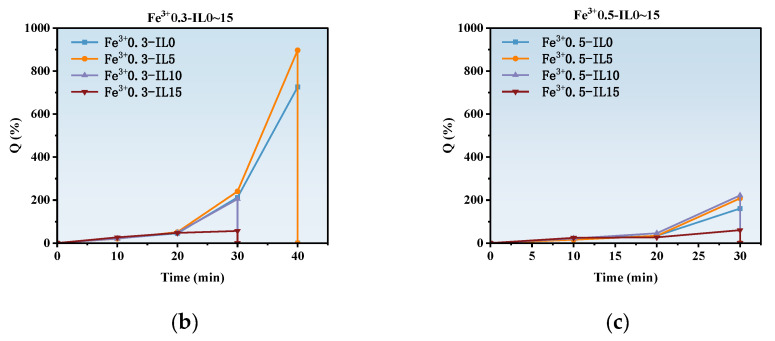
(**a**) Photos of the ionohydrogel after being placed in water for a certain amount of time. (**b**) Dissolution rate of the ionohydrogel with IL content at a fixed Fe^3+^ concentration of 0.3 mol%. (**c**) Dissolution rate of the ionohydrogel with IL concentration at a fixed Fe^3+^ concentration of 0.5 mol%.

The PAA-Fe^3+^-IL MBN ionohydrogel is soaked in deionized water, as shown in Figure 7a. It dissolves after two hours later. This is due to the physical crosslinking are far more than the chemical crosslinking of metal coordination crosslinking in the ionohydrogel. These metal coordination crosslinks can be destroyed, leading the ionohydrogel to dissolve.

When the Fe^3+^ concentration is 0.3 mol%, the water absorption with time curve of the PAA-Fe^3+^-IL MBN ionohydrogel is obtained, as shown in Figure 7b (horizontal coordinates indicate time, vertical coordinates indicate water absorption, and light blue line, orange line, purple line, and dark red line represent the IL contents, which are 0 wt%, 5 wt%, 10 wt%, and 15 wt%). It can be seen that a water-soluble phenomenon occurs after 20 min with the IL content of 10 wt%~15 wt%. Whereas, the water-soluble phenomenon occurs after 40 min with the IL content is 0 wt%~5 wt%. As the IL content increases, the number of metal coordination crosslinks increases. Thus, the water-soluble phenomenon occurs earlier. The ionohydrogels with Fe^3+^ concentrations of 0.05 mol% and 0.1 mol% are directly degraded in water.

When the concentration of Fe^3+^ is 0.5 mol%, the water absorption rate of the PAA-Fe^3+^-IL MBN ionohydrogel varies over time, as shown in Figure 7c (horizontal coordinates indicate time, vertical coordinates indicate water absorption rate, and light blue line, orange line, purple line, and dark red line indicate the contents of IL, which are 0 wt%, 5 wt%, 10 wt%, and 15 wt%). When the Fe^3+^ concentration is 0.5 mol%, the water-soluble phenomenon of the ionohydrogel needs 30 min. The water absorption rate increases with increasing the IL content when the the IL content is 0 wt%~10 wt%. Whereas, the water absorption rate drops sharply to about 50% of the last situation with the IL content of 15 wt%.

By comparing the data in Figure 7b and 7c, it can be seen that the MBN ionohydrogel containing more ferric ions has a lower water absorption expansion rate at a certain time point. This is because the metal coordination bonds formed by the ferric ions are relatively weak and easily destroyed in water by swelling. The water-soluble phenomenon is conducive to the degradation of the ionohydrogel.

### 3.5. Humidity Sensing of PAA-Fe^3+^-IL Ionohydrogels

**Figure 8:**
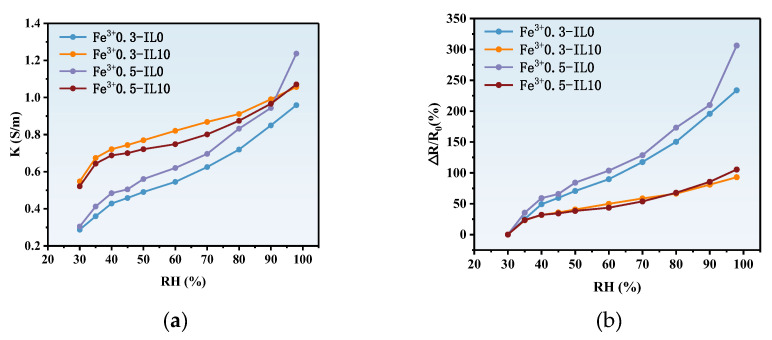
(**a**) Conductivity of PAA-Fe^3+^-IL ionohydrogels with different Fe^3+^ concentrations and different IL contents at different humidity levels. (**b**) Electrical resistance variability of PAA-Fe^3+^-IL ionohydrogels with different Fe^3+^ concentrations and different IL contents at different humidity levels.

Due to the excellent water absorption properties of the ionohydrogel, a humidity sensing test was performed in this study. The high-conductivity ionohydrogel was placed in a humidity box at high and low temperatures. By controlling the relative humidity of the box, the ionohydrogels presented different conductivities, as shown in Figure 8a. The conductivity of the ionohydrogel increased with increasing relative humidity.

The conductivity calculation formula used in Figure 8 is shown in Formula (3) above. The resistance change rate is also used as a factor to evaluate the humidity sensing performance of ionohydrogels, as shown in Formula (9):

(9) $$∆R/R_{0}=(R-R_{0})/R_{0}\times 100\%$$

where R_0_ is the initial resistance of the ionohydrogel samples and R is the resistance at a certain strain.

When the IL content of the ionohydrogel is fixed, with increasing humidity, the conductivity is increased with different ferric ion concentrations. When the metal ion concentration is kept the same, a higher content of IL means that the ionohydrogel has a higher conductivity with increasing relative humidity. An increase in ferric ions gives the ionohydrogel a higher resistance rate, as shown in Figure 8b. At the same relative humidity, for example, when the IL content was 10 wt%, the conductivity was higher than for the ionohydrogel without added IL. This is due to the fact that IL is a hydrophilic ionic liquid that can increase the conductivity of the ionohydrogel by absorbing water. The ionohydrogel in this study provides ideas for the future of electronic skin.

## 4. Conclusions

This work presents the development of recoverable ionohydrogels with enhanced mechanical and conductive properties through the introduction of 1-ethyl-3-methylimidazole acetate (IL) and ferric ions into polyacrylic acid (PAA) ionohydrogels. The ionohydrogels were synthesized using free radical polymerization, resulting in a multi-bond network (MBN) structure that significantly improved their toughness and conductivity. The obtained PAA-Fe^3+^-IL ionohydrogels showed a high tensile strength of 495.09 kPa and a large elongation at break of 1151.35%. With the existence of large quantities of mobile ions from IL and Fe^3+^, the ionohydrogels displayed a superior conductivity of 1.48 S/m and a high strain sensitivity with a GF of 8.04. The tensile behavior of the ionohydrogels was analyzed by means of the viscoelastic model proposed by Sparkon et al. Most hydrogels are difficult to use for a long time due to their loss of water [60]. Ionohydrogels have very good water retention capabilities to make up for this defect. Moreover, the physical crosslinking density of this ionohydrogel is much higher than its chemical crosslinking density, which makes it biodegradable. PAA-Fe^3+^-IL ionohydrogels with fast water absorption capabilities can be used for moisture absorption due to this linear water absorption response. They can also be used for humidity sensing. These tough and conductive PAA-Fe^3+^-IL ionohydrogels with multiple functions provide new ideas for applications in intelligent and wearable electronic products.

## Data Availability

The original contributions presented in this study are included in the article. Further inquiries can be directed to the corresponding authors.
